# Reversible cerebral vasoconstriction syndrome with cerebral infarction caused by acute high-level vapor exposure of ethylene oxide: a case report

**DOI:** 10.1186/s12883-021-02429-9

**Published:** 2021-10-09

**Authors:** Bin Lin, Chao Wang, Nan Lu, Le Zhang, Biao Jiang

**Affiliations:** 1 Department of Radiology, The Second Affiliated Hospital, Zhejiang University School of Medicine, No.88 Jiefang Road, Hangzhou, 310009 Zhejiang China; 2 Department of TCM Rehabilitation, The Second Affiliated Hospital, Zhejiang University School of Medicine, Hangzhou, China

**Keywords:** Ethylene oxide, Toxicology, Encephalopathy, Infarction, Headache

## Abstract

**Background:**

With the increasing production and use of ethylene oxide (EO) worldwide, its explicit bio-toxicity has drawn more and more attention. At present, most studies focus on chronic EO exposure. Studies on acute EO exposure are rare, especially with imaging studies. To our knowledge, this work is the first documented case of reversible cerebral vasoconstriction syndrome (RCVS) with cerebral infarction caused by EO.

**Case presentation:**

A 58-year-old woman who worked in a capsule production factory got an unprotected acute EO inhalation due to accidental exposure to sterilization gas. She suffered from nausea, vomiting, and severe paroxysmal headaches, but the first brain MRI scan of the patient showed no significant abnormality. Nine days after inhalation, she developed recurrent thunderclap headaches and gradual complete blindness. The follow-up brain MRI, 12 days after inhalation, demonstrated extensive cytotoxic edema. Fifteen days and 21 days after EO (ethylene oxide) inhalation, head MRA and CTA respectively showed diffuse vasoconstriction of cerebral arteries. Fifty-nine days after EO inhalation, head MRA assessed reversibility of the vasoconstriction. According to clinical features and imaging findings, RCVS with cerebral infarction can be diagnosed. The patient was sensitive to light and light reflection but still blind after symptomatic and rehabilitation therapy.

**Conclusions:**

We report an acute EO exposure case in which the patient suffered from RCVS with cerebral infarction, which previous literature has not reported. This article aimed to raise awareness of encephalopathy after EO acute exposure.

## Background

Ethylene oxide (EO) is a colorless gas at room temperature with a pungent smell and colorless transparent liquid at about 12 degrees Celsius (53 degrees Fahrenheit). EO is an essential intermediate product in industrial production and a highly effective sterilizer at low temperatures. However, with the increasing production and use of EO worldwide, its explicit bio-toxicity has drawn more and more attention [1]. After inhalation or ingestion, the dissolved EO in the blood can often be metabolized and excreted within 24 h [2]. However, high levels of EO exposure can overwhelm the metabolic process and result in EO accumulation and its by-products resulting in toxic effects [3]. Symptoms in previously reported cases of EO acute exposure include dizziness, pulsatile headache, nausea, emesis, fatigue, paresthesia, drowsiness, ataxia, confusion, convulsions, mucous membrane irritation in eyes, nose, and respiratory tract, contact dermatitis and burns, some of which showing allergic reaction in type I or type IV with various clinical symptoms and even lead to death [4]. EO acute exposure is mainly caused by leakage due to operating procedures or unprotected operation [1]. At present, most studies focus on chronic EO exposure. There have not been many studies on EO acute exposure, especially those with diagnostic imaging that goes unreported.

## Case presentation

**Fig. 1:**
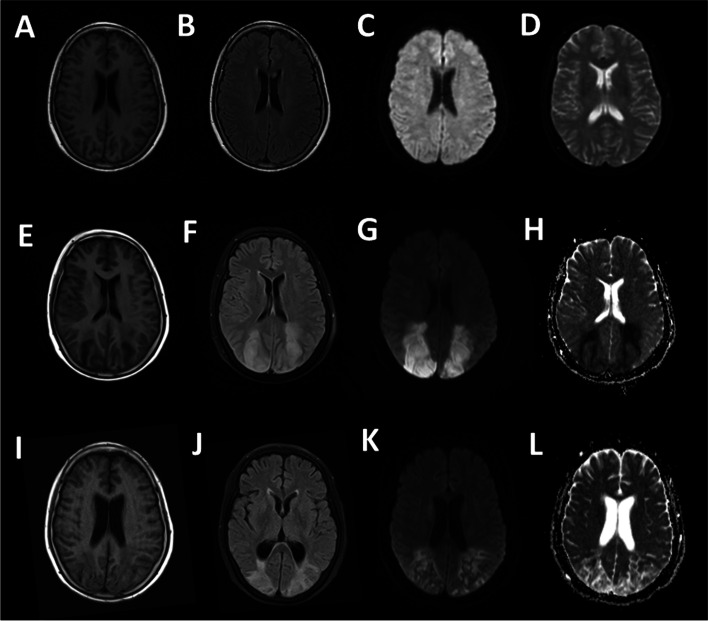
Initial and follow-up axial T1WI, FLAIR, DWI, and ADC brain MRI. **A**-**D**: Axial T1WI, FLAIR, DWI, and ADC brain MRI performed 5 days after inhalation indicated that there was no noticeable abnormality. **E-H**: Axial T1WI, FLAIR, DWI, and ADC brain MRI performed 12 days after exposure demonstrates extensive cytotoxic edema in the parietal and occipital lobes bilaterally, left frontal lobe, centrum semiovale region. **I-L**: Axial T1WI, FLAIR, DWI, and ADC brain MRI performed 59 days after exposure demonstrates a notable improvement of the intracranial arterial stenosis

**Fig. 2:**
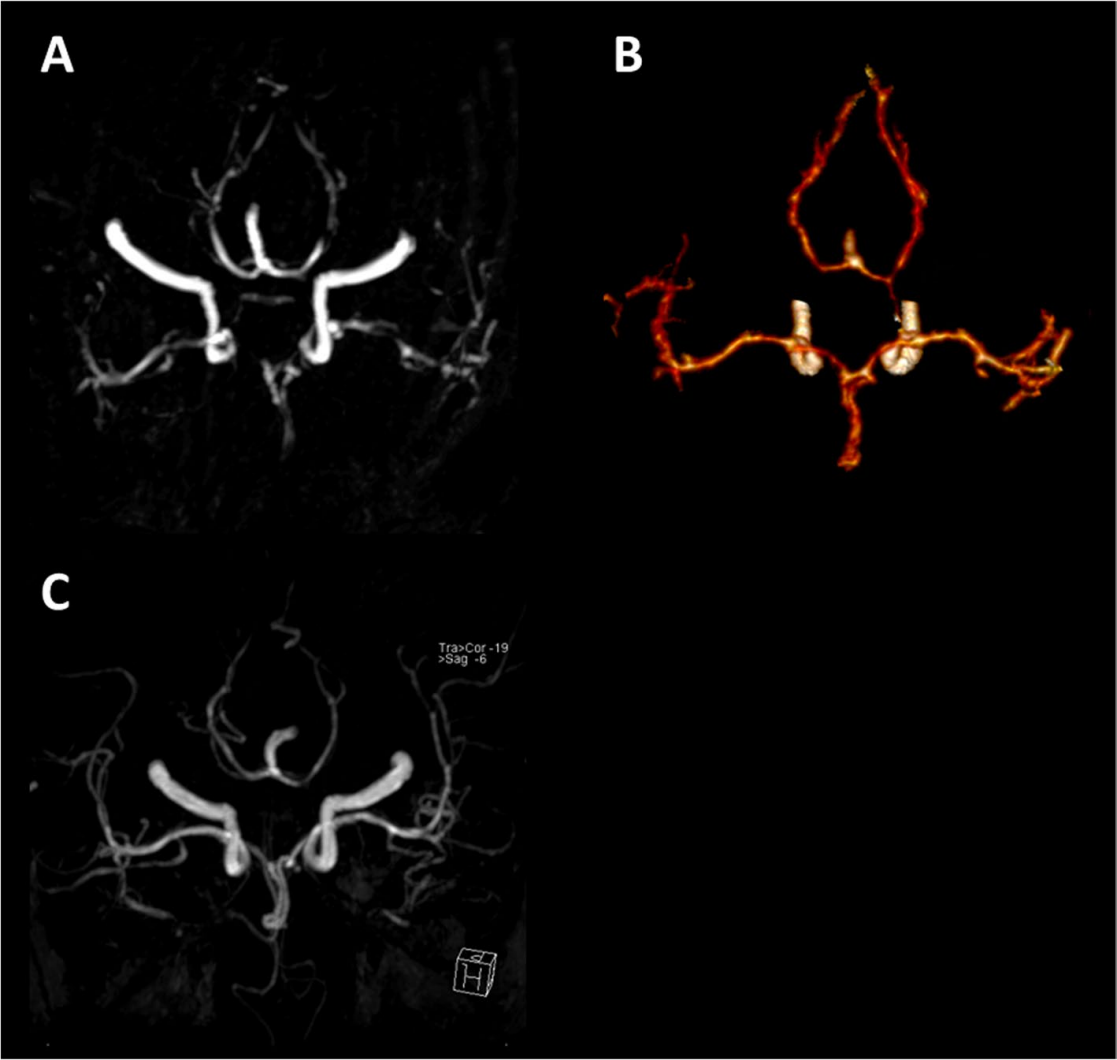
Initial and follow-up CTA and MRA. The 15 days and 21 days after ethylene oxide inhalation, Head MRA (**A**) and CTA (**B**) showed multiple areas of stenosis and partial occlusions involving the anterior, medial and posterior cerebral arteries bilaterally, respectively. The 59 days after ethylene oxide inhalation, the follow-up head MRA (**C**) showed that the cerebral arterial stenosis was markedly improved but not complete recovery

A 58-year-old right-handed woman was a sterilization worker in a capsule production factory with nearly 10 years of service length. She suffered from nausea and vomiting after unprotected acute EO inhalation of leaked sterilization gas containing 75% EO and 25% carbon dioxide in an air-locked room for an hour. The indoor concentrations of EO were uncertain. She had severe paroxysmal headaches, which were relieved when she came back home. The brain MRI of our hospital scan showed no abnormality 5 days after inhalation (Fig. 1, panel A-D). Nine days after inhalation, the patient developed recurrent thunderclap headache (striking suddenly like a clap of thunder and the severe headaches peaks within 60 s) and partially lost bilateral eyesight in the whole field, which progressed to complete blindness and no light perception over the next 24 h. A brain MRI scan performed again after admission, 12 days after inhalation, demonstrated extensive cytotoxic edema in the bilateral parietal lobe, occipital lobe, and left frontal lobe (Fig. 1, panel E-H). Fifteen days and 21 days after inhalation, Head MRA and CTA showed multiple areas of stenosis and partial occlusions involving the anterior, medial and posterior cerebral arteries bilaterally, respectively (Fig. 2). Laboratory tests: 12 days after inhalation, D-Dimer 880 μg/L(FEU)↑, GLB - 31.6 g/L↑, TG - 3.12 mmol/L↑. Subsequently, D-Dimer was continuously tested, this index through the process from rising to falling. 17, 32, 41, 51, 54 days after EO inhalation, D-Dimer value was 2920, 2500, 4960, 1630, and 760 μg/L(FEU)↑, respectively. Pupillary light reflex was normal. The patient was alert and oriented to person, place, time, situation but slow to respond to questions. The patient had no history of high blood pressure, diabetes, hyperlipidemia, cerebrovascular disease. She did not drink alcohol or smoke and was without a family history of the inheritable disease. Currently, no consensus on the cut-off point for acute and chronic EO exposure. According to most definitions of intoxication, acute exposure is usually within 24 or 72 h of poisoning. Thus, the patient admitted with a presumptive diagnosis of “encephalopathy caused by EO acute exposure” because the patient developed clinical symptoms about 1 h after EO exposure.

During the hospitalization, the patient presented with left-sided hemiparesis and cortical blindness. After more than 2 months of symptomatic and rehabilitation therapy, the patient was sensitive to light but still blind after the therapy. Subsequently, the cerebral arterial stenosis markedly improved on a follow-up head MRA 59 days after inhalation (Fig. 2, panel C). Moreover, the DWI and ADC showed the lesions turned into softening lesions (Fig. 1, panel I-L).

## Discussion

With the widespread use of ethylene oxide worldwide, its biotoxicity has attracted more and more attention. Ethylene oxide (EO) reacts with nearly all cellular components, including proteins, vitamins, cofactors, DNA, and RNA. Clinically, EO poisoning could cause acute neurological symptoms, including persistent nausea, vomiting, and recurrent thunderclap headache [5]. For the first time, we reported the case of reversible cerebral vasoconstriction syndrome (RCVS) with cerebral infarction caused by EO [6]. Specifically, the first-time brain MRI scan of the patient showed no significant abnormality. Next, 15 days and 21 days after EO inhalation, head MRA and CTA respectively showed diffuse vasoconstriction of cerebral arteries. Finally, 59 days after EO exposure, MRA assessed reversibility of the vasoconstriction was performed. According to clinical features and imaging findings, the diagnosis of reversible cerebral vasoconstriction syndrome (RCVS) is thus made. The patient did not receive any drug treatment with the nine-day intervals between EO exposure and recurrent thunderclap headache at onset and had no known exposure to other vasoconstrictors associated with RCVS. The patient’s RCVS_2_ score was 7, with a score of > 5 having high specificity and sensitivity (99 and 90%, respectively) for diagnosing RCVS (7). RCVS caused cerebral watershed infarction and posterior circulation infraction the brain MRI 12 days after inhalation showed. However, RCVS should be differentiated from another reversible neurological disease, the posterior reversible encephalopathy syndrome (PRES). These two diseases share many clinicoradiographic features, suggesting overlapping or similar pathophysiological mechanisms [5]. The PRES rarely causes stroke, although cerebral infarction, cytotoxic edema can occur in some cases [7]. The diagnosis of RCVS may be more reasonable. The imaging findings did, however, differ from other types of acute inhalation intoxication. Unlike toxic encephalopathy caused by other organic solvents and toxic gas inhalation, EO exposure does not involve the basal ganglia region, thalamus, dental nucleus, or cortical grey matter. It does not produce typical vasogenic cerebral edema or demyelination [8]. However, it may be similar to acute inhalation of vasoactive drugs such as cocaine which can cause RCVS [9].

Owing to its broad-spectrum antibacterial activity, EO is also used to sterilise hospital equipment and supplies that are not resistant to high temperature or high humidity, such as optical instruments, precision appliances, electronic components, and plastic capsulation. However, EO is a genotoxin and carcinogen with strong biotoxicity [10]. EO was recognized as one of the first carcinogens by the International Agency for Research on Cancer, an agency under the World Health Organization in 2017, and a hazardous air pollutant by the United States Environmental Protection Agency in November 2019.

## Conclusions

The central nervous system is one of the primary targets of EO exposure, although the mechanism of neurotoxicity remains unclear. Finelli et al. postulated that EO might interfere with the metabolism of neuronal perikaryon or axonal transport, thus inhibiting the delivery of essential metabolites to nerve terminals [11]. RCVS from EO exposure has not been reported before. Some experimental animal studies are showing that EO exposure can cause vascular damage [12, 13]. We speculate that the high concentration of EO inhalation caused vascular damage cerebral arteries of this patient and ultimately led to RCVS. Further research into the mechanism of injury on the central nervous system of acute EO exposure is necessary to determine potential vasoconstrictive effects on cerebral arteries and choose the optimal treatment for exposed patients.

## Data Availability

All data related to this case report are documented within this manuscript.

## Abbreviations

EO: Ethylene oxide
RCVS: Reversible cerebral vasoconstriction syndrome
CT: Computed tomography
PRES: Posterior reversible encephalopathy syndrome
CTA: Computed tomography angiography
MRI: Magnetic resonance imaging
MRA: Magnetic resonance angiography
